# How to Screen for Non-Adherence to Antihypertensive Therapy

**DOI:** 10.1007/s11906-016-0697-7

**Published:** 2016-11-26

**Authors:** Pankaj Gupta, Prashanth Patel, Robert Horne, Heather Buchanan, Bryan Williams, Maciej Tomaszewski

**Affiliations:** 1 Department of Metabolic Medicine and Chemical Pathology, University Hospitals of Leicester NHS Trust, Leicester, UK; 2 National Institute of Health Research Leicester Cardiovascular Biomedical Research Unit, Glenfield Hospital, Leicester, UK; 3 British Heart Foundation Cardiovascular Research Centre, Department of Cardiovascular Sciences, University of Leicester, Leicester, UK; 4 Department of Behavioral Medicine, School of Pharmacy, University College London, London, UK; 5 Division of Rehabilitation and Ageing, School of Medicine, University of Nottingham, Nottingham, UK; 6 Institute of Cardiovascular Science, University College London, London, UK; 7 National Institute for Health Research University College London Hospitals Biomedical Research Centre, London, UK; 8 Division of Cardiovascular Sciences, Faculty of Biology, Medicine and Health, University of Manchester, Manchester, UK; 9 Division of Medicine, Central Manchester NHS Foundation Trust, Manchester Academic Health Science Centre, Manchester, UK

**Keywords:** Hypertension, Uncontrolled hypertension, Non-adherence, Blood pressure medication

## Abstract

The quality of assessment of non-adherence to treatment in hypertensive is poor. Within this review, we discuss the different methods used to assess adherence to blood-pressure-lowering medications in hypertension patients. Subjective reports such as physicians’ perceptions are inaccurate, and questionnaires completed by patients tend to overreport adherence and show a low diagnostic specificity. Indirect objective methods such as pharmacy database records can be useful, but they are limited by the robustness of the recorded data. Electronic medication monitoring devices are accurate but usually track adherence to only a single medication and can be expensive. Overall, the fundamental issue with indirect objective measures is that they do not fully confirm ingestion of antihypertensive medications. Detection of antihypertensive medications in body fluids using liquid chromatography–tandem mass spectrometry is currently, in our view, the most robust and clinically useful method to assess non-adherence to blood-pressure-lowering treatment. It is particularly helpful in patients presenting with resistant, refractory or uncontrolled hypertension despite the optimal therapy. We recommend using this diagnostic strategy to detect non-adherence alongside a no-blame approach tailoring support to address the perceptions (e.g. beliefs about the illness and treatment) and practicalities (e.g. capability and resources) influencing motivation and ability to adhere.

## Introduction

Hypertension affects more than a billion patients worldwide and is the leading cause of global disease burden, ahead of smoking and obesity [1]. High blood pressure (BP) is one of the most important modifiable risk factors of cardiovascular disease. Although potent antihypertensive medications are available, BP is optimally controlled in only half to two thirds of patients [2, 3]. One potential explanation for such a low rate of BP control is that antihypertensive medications are not taken as prescribed or not taken at all [4–6]. Non-adherence is defined as ‘the extent to which a person’s behaviour—taking medication, following a diet, and/or executing lifestyle changes, corresponds with agreed recommendations from a health care provider’ [7•]. Non-adherence to antihypertensive therapy is known to correlate with poor cardiovascular outcomes. It is the main reason for treatment failure and repeated hospital admissions [8]. Conversely, good adherence to blood-pressure -lowering therapy is associated with a reduction of adverse cardiovascular outcomes [9•]. Unfortunately, up to 40% of clinical appointments fail to address adherence [10, 11•]. Furthermore, patients are frequently reluctant to volunteer information about suboptimal adherence to treatment [12].

Resistant hypertension is defined as uncontrolled BP despite the use of three different classes of antihypertensive therapy at maximally tolerated doses, one of which is a diuretic [13]. The prevalence of resistant hypertension is estimated at 10–20% in patients with elevated BP [13–16]. Patients with resistant hypertension have a higher incidence of cardiovascular disease and mortality than the general hypertensive population [13]. Without an appropriate diagnostic approach, resistant hypertension is often difficult to differentiate from pseudo-resistant hypertension (apparent resistant hypertension) [14]. The latter is usually caused by white coat effect/hypertension, suboptimal choice/doses of antihypertensive medications and/or non-adherence to treatment [17]. Our recent analysis suggests that non-adherence is the dominant type of pseudo-resistant hypertension in patients referred for renal denervation [18•]. Thus, addressing non-adherence is of critical importance in the management of patients diagnosed with resistant hypertension [6, 19].

## How to Diagnose Non-Adherence

### Subjective Methods

#### Physician Perception and Patient Self-Reported Methods

Research has demonstrated that physicians are generally not good judges of whether their patients are taking their medication as prescribed. Indeed, physicians’ perception of non-adherence correlated with an objective measure in less than 40% of cases. Moreover, 40% of diagnoses of non-adherence based on clinical judgment results in treatment escalation that through augmentation of polypharmacy may further compromise adherence [20]. Increasing the number/doses of antihypertensive medications despite an impression of non-adherence implies that the confidence of physicians in the diagnostic capacity of their perception is poor. Hence, this method is not useful in diagnosing non-adherence in clinical practice.

Asking patients to report on their own medication adherence also leads to inaccurate estimates. A number of questionnaires available to diagnose adherence are easy to implement, cheap and widely available [21]. Of around 20 different patient self-reported questionnaires validated to some extent for use in hypertension, the four-item Morisky Adherence Questionnaire (MAQ) is perhaps the most commonly used. The expanded version of Morisky Medication Adherence Scale (MMAS), Hill–Bone Compliance Scale, Medication adherence questionnaire and Medication Adherence Self Efficacy Scale (MEMS) are also used [22•]. However, their internal validity varies (Cronbachs’s α 0.61–0.91) and their specificity is generally below 75%. Adherence is overreported by up to 20% when compared to an objective measure [23]. These overestimates may be due to a number of reasons, for example social desirability (wanting to be seen as a ‘good’ patient). Thus, questionnaires have an advantage of providing some insights into barriers of non-adherence [7•] but are not associated with prediction of cardiovascular outcomes [9•, 24]. Due to the limitations highlighted above, questionnaires are not competitive against objective methods of diagnosing non-adherence .

### Objective Methods

#### Indirect Methods

##### Review of Pharmacy Database Records

Acquisition of medications by patients is considered as a surrogate of adherence to treatment. The most common type of this measure is medical possession ratio (MPR) defined as the number of days of medication supplied to the number of days in the observation multiplied by 100 [25]. MPR >80% is generally considered as the threshold of good adherence as there is some evidence that it predicts future hospitalisation [26]. Pharmacy database records are a popular method of assessing adherence; they can provide some insights into various components of non-adherence including persistence and discontinuation rates [27•, 28–32]. The prevalence rates of non-adherence assessed through review of pharmacy database records range between 14 and 76%; the association with increased risk of cardiovascular outcomes varies [27•, 28–32]. A high variation may reflect (at least in part) inherent limitations of this method in diagnosing non-adherence. For example, the accuracy of non-adherence detected also depends on the completeness and robustness of the records. A systematic review of pharmacy patient records has revealed that significant medication errors were noted in all studies [33]. There was an error rate of between 13 and 29% in the retention of discontinued medications on the records, and 24% of medication lists errors were classified as significant [33]. The accuracy of adherence assessed using pharmacy records also depends on patients having access to only monitored systems of medications supply and the integration of data across the monitored systems of prescription refill. The correlation between pharmacy refill records and an objective measure of testing for non-adherence (electronic monitoring) is poor [34]. Furthermore, retrospective pharmacy records may not be reflective of a patient’s current disease condition, medication taking pattern or behaviour. Finally, obtaining information from pharmacy records may be difficult where primary health care records are not integrated across various systems/providers and electronic records are not ubiquitously used.

##### Pill Counting

This indirect measure of adherence relies on counting the number of pills that remain with a patient after a prescribed period. Pill-counting overestimates non-adherence by roughly 8% when compared to data from objective electronic monitoring devices [23]. Although in theory it is a simple method, it is entirely dependent on the patient’s cooperation (bringing their pill containers to the clinic), it does not take into account any surplus medication the patient may have from previous prescriptions, and it is time consuming in a busy clinical setting [21]. It is also often difficult to determine the baseline number of pills with a patient and is open to inadvertent omission due to pills being stored in different places [35]. Patients may also remove pills when a clinic visit is due in order to mask non-adherence. Hence, this method is not commonly used to assess non-adherence in clinical practice.

##### Electronic Monitoring Devices

Electronic monitoring devices (EMDs) can be broadly divided into devices that record adherence and those that also provide feedback to the patient and/or the health care provider. They range from simple audiovisual alarm devices, digital recorders with display, to sophisticated devices which use pill dispensers with electronic sensors activated by the act of opening. Feedback may be provided in the form of audiovisual alerts, prompts on computer systems or wirelessly through text messaging and mobile phones. These data can be transmitted to the patient, their relatives, their health care provider or support care providers [36•]. Medication event monitoring systems (MEMS) is the most widely used EMD; it provides a rich granularity of data on dosing times, patterns of non-adherence including lack of initiation of medication, persistence and discontinuation periods, and periods of good and poor adherence [37]. It has been widely used in research studies [38•]. There is limited data available on the ability of non-adherence detected in EMDs to predict adverse cardiovascular outcomes. The recent meta-analysis of prospective epidemiological studies on non-adherence to cardiovascular therapy included one small study that used an EMD [9•]. The study demonstrated that the likelihood of adverse outcomes was decreased in patients with heart failure who had good adherence [39]. However, EMDs are expensive and each device can currently monitor only one medication. Therefore, it is difficult to assess overall non-adherence in patients on multiple medications such as those with resistant hypertension. Recently, multi-compartment pill organisers with electronic monitoring have become available [40]. There are also adhesive labels or thin polymers with sensors available to attach on blister packs [30, 41]. These polypharmacy electronic monitoring systems (POEMS) overcome the limitation of single medication monitoring.

EMDs may undergo mechanical failure, and patients may take out more than one pill with each opening [42]. There is also an increased adherence rate reported on initial use of the devices; thus, EMDs may to some extent act as intervention in increasing adherence to medication [35]. Patients often take their medications outside of their home, and thus, the systems may be bulky and intrusive to the patient’s privacy [40]. Despite these limitations, EMDs are probably one of the best indirect objective methods of diagnosing non-adherence [23]. The main issue remains the high cost of the devices and the fact that device opening does not equate to ingestion of pills.

#### Direct Methods

Direct methods include directly observed therapy, digital pills and biochemical measurements in urine or blood.

##### Directly Observed Therapy

Directly observed therapy (DOT) clinics invite patients to attend the hospital and take part in sequential ingestion of antihypertensive mediations at intervals of 1–2 h under direct continuous observation of a nurse. Twenty-five percent of non-adherent patients were reported to develop symptomatic hypotension when assessed using this method [43]. There are also anecdotal reports of non-adherent patients being admitted to intensive care due to a precipitous drop in blood pressure driven by ingestion of previously avoided prescribed medications. Apart from safety concerns, there are other limitations of this method including costs, logistics and labour (the patients usually have to come for at least half-a day and be supervised by trained staff).

##### Digital Pills

A recent innovation is the development of pills with ingestible sensors that emit a signal when activated by gastric juices. The signal is detected by a patch worn by the patient, and the information remotely transmitted to a health care provider. Digital pills have been shown to be effective where a course of medications is needed for a limited timeframe such as in tuberculosis [44]. Although these have been approved for use by the FDA and European Medicines Agency, there is as yet only a limited uptake of this technique. It is also not clear how acceptable this technique might be to patients.

##### Biochemical Detection of Medications in Urine or Blood

We and others have recently developed an objective method to assess non-adherence in bodily fluid samples [38•, 41, 45–50]. The method utilises high performance chromatography-tandem mass spectrometry (HPLC-MS/MS) which is commonly used in forensic toxicological analysis [51, 52]. HPLC-MS/MS-based analysis requires a simple urine/blood sample. A total of 5–10 ml of urine collected in a plain container, frozen and analysed in batches. Our assay tests for the presence/absence of 40 most commonly prescribed antihypertensive medications [38•]. The non-detection of expected antihypertensive medication in urine is consistent with non-adherence to the prescribed antihypertensive lasting at least as long as its four half-lives [53]. This time is different for each antihypertensive medication dependent on their pharmacokinetic profile. For a majority of antihypertensive medications, this time is in excess of 24 h. For example, the absence of amlodipine in urine (half-lives, 35–70 h; 4 half-lives, 140–280 h) means that the medication was not taken for at least 5.8 days prior to the urine sample collection [53].

HPLC-MS/MS instrumentation is expensive (≈$250,000–$300,000) and requires skilled laboratory staff. These resources are available in most teaching hospitals in the UK and the same would be true in similar centres across the Western world. The analytes are stable in urine [50], and hence, we envisage a model of non-adherence testing with regional centres where samples could be sent for analysis by HPLC-MS/MS. Indeed, our laboratory receives samples from across 20 centres in the UK and has analysed more than 2000 samples to date. A recent predictive modelling study demonstrated that repeated biochemical screening for non-adherence to antihypertensive treatment (known as therapeutic drug monitoring) is cost-effective in the management of resistant hypertension [54].

Biochemical assessment by HPLC-MS/MS provides only a snapshot of non-adherent behaviour. The detection of a prescribed antihypertensive medication in blood/urine does not equate to persistence. Furthermore, the HPLC-MS/MS-based urine analysis is not immune to “tooth-brush adherence” or white-coat adherence—sudden improvement of adherence prior to a clinic visit (similar to the behaviour of patients brushing their teeth before visiting a dentist) [55].

## When to Assess for Non-Adherence

Given the high incidence of non-adherence in patients presenting with ‘resistant hypertension’, we would suggest screening for non-adherence in all such patients [56•]. We have demonstrated that approximately one in three patients referred for renal denervation was non-adherent to their antihypertensive treatment [18•]. The recent data from DENERHTN trial suggest that non-adherence is even more common amongst patients in whom renal denervation was conducted [57]. Thus, it is worth considering screening for non-adherence in patients with resistant hypertension prior to irreversible and expensive interventions such as renal denervation.

There is a subgroup of patients who are considered to have refractory hypertension. This is defined as patients who have uncontrolled blood pressure despite being on ≥5 antihypertensive medications (usually on two diuretics) and under specialist care for their hypertension [58]. The prevalence is thought to be between 3 and 10% of patients referred with uncontrolled resistant hypertension to a specialist clinic [59, 60]. The prevalence of refractory hypertension is estimated at 0.5% of all hypertensives [13]. We suggest excluding non-adherence to BP-lowering therapy by an objective method in all such patients before classifying them as truly refractory to antihypertensive medications.

In our experience, the overwhelming number of patients on monotherapy are likely to be adherent and therefore, non-adherence testing is unlikely to be of benefit or cost-effective in this group of patients. Conversely as the number of medications increase, it is more likely that patients are non-adherent. Therefore, it would be useful to test for non-adherence if there is a lack of an expected response in blood pressure in patients on optimal antihypertensive therapy, even if they do not satisfy the definition of resistant hypertension.

## Which Interventions Improve Non-Adherence?

A Cochrane review of interventions with a potential to improve non-adherence was published in 2012 [61•]. Only 13 studies related to non-adherence to antihypertensive treatment were of sufficient quality to be included in this review. Of these, four used self-reported measures of non-adherence, five used pill counts and the remaining four used MEMS to monitor non-adherence. Adherence rates improved by 3% (pill counts) to 36% (self-report) in 11 of the 13 studies. Systolic BP improved by 3–9.5 mmHg in seven studies, and in two studies, only diastolic BP improved by 3–4 mmHg [61•]. There were seven studies that showed improved adherence and a significant change in BP. The interventions that were of benefit were complex and included various combinations of detailed patient education or discussion, simplifying dose regime, patient-reported BP measurements, telephone discussions and lifestyle advice [61•]. The findings of the review are in keeping with the WHO view that non-adherence needs to be considered in a holistic manner [7•]. All barriers such as difficulty in access to health care, cost of medications, lack of social support, inadequate health care provider, patient education (especially for asymptomatic diseases such as hypertension), lack of training of health care providers, complexities of medication dosing and co-morbidities including depression need to be addressed [7•]. Furthermore, any intervention needs to be underpinned by a tailored approach, that is focused on a patient’s concerns and beliefs about hypertension and medications is formulated [62•]. The Cochrane review concluded that overall, the quality of research was poor and emphasised the need to use objective measures of non-adherence in future research in this field [61•].

## Our Clinical Experience

We have been using urine HPLC–MS/MS analysis in routine clinical practice for the last 4 years. We collect urine samples from the patients with their prior consent on the day of their clinic visit. The results of the analysis are then discussed at the subsequent appointment. Through its unbiased nature, the urine analysis provides the physicians with necessary confidence to discuss non-adherence and its causes. Such discussions are conducted in a non-confrontational and a non-judgmental manner further to the biochemical confirmation of the non-adherence to antihypertensive treatment. We explain to patients that the lack of medications in the body is the reason why their blood pressure is high. This simple linkage, in our experience, often changes the perception of patients about the benefit of medications in controlling their hypertension. We also address patient beliefs about medications. A key misconception is that because there are no symptoms of the disease (hypertension is mostly asymptomatic) and/or there is actual improvement in well-being, the persistence with antihypertensive medications is assumed to be unnecessary [7•, 62•]. Common reasons of non-adherence [7•] such as forgetfulness and polypharmacy are also addressed, and simple cost-effective solutions such as a pill organiser, reminder techniques and/or the reduction in the number of prescribed medications (combination pills) are suggested. The HPLC-MS/MS-based urine analysis is performed on follow-up appointments.

## The Ethical, Clinical and Economic Aspects of Screening for Non-Adherence to Antihypertensive Treatment

There may be concerns that objective testing is similar to ‘policing’ the patients and reflects a disciplinary society where hierarchical organisations have the dominant power and individuals lose control of their self and their privacy [63]. The alternative view is that such monitoring (especially digital) is part of information society that individuals are willing to be part of and thus gain personal benefit [64]. From the clinical point of view, ignoring or ineffective way of testing for non-adherence will result in unnecessary treatment escalations and additional investigations, many of which carry risks. From the health economy point of view, the consequences of ineffective diagnostic approaches to non-adherence to antihypertensive treatment are extremely expensive reaching approximately $1000 to $1500 per patient in the UK. Improvement in non-adherence rates in hypertensive patients by 25% can lead to reduction of adverse events by more than 2 million and lead to savings of $20billion [65]. On the individual level, detecting non-adherence objectively prevents unnecessary investigations, hospital visits and helps bringing to the surface the patient’s views about medications and their understanding of their illness which can then be discussed openly in a patient-centred manner. In our experience, patients are very rarely concerned about the urinary screening for adherence. Anecdotally, we have had similar feedbacks from other clinicians across UK who use our service to detect non-adherence by HPLC-MS/MS.

## Conclusion

Non-adherence to BP-lowering therapy is common in patients with uncontrolled hypertension and is much higher in patients with ‘resistant hypertension’ than in the general hypertensive population. Therefore, testing for non-adherence in these patients should become a part of routine clinical practice. In our view, subjective methods to assess non-adherence such as patients’ reports should be used with caution. Objective measures are more reliable but are more expensive. Amongst the indirect objective measures, EMDs with patient feedback provided by a health care provider are to be preferred over pharmacy records but the usage would be dictated by resources. Some direct methods such as DOT are expensive and can lead to hypotensive events. In our view, the biochemical analysis by HPLC-MS/MS, if available, is the most accurate and practical technique for use in busy clinics. Due to the non-invasive nature of collection of urine, the latter is preferred to blood. The confirmation of non-adherence to antihypertensive treatment should be followed by appropriate management centred on a Perceptions and Practicalities Approach to adherence support endorsed by the NICE Medicine Adherence Guidelines [12]. Ultimately, achieving sustained adherence is likely to require a no-blame approach facilitating a Perceptions and Practicalities Approach to adherence support endorsed by the NICE Medicine Adherence Guidelines [12]. Ultimately, achieving sustained adherence is likely to require a no-blame approach facilitating an open discussion to identify and address the perceptions (e.g. beliefs about illness and treatment) and practicalities (e.g. capability and resources) influencing the patient's motivation and ability to adhere to antihypertensive treatment. 
